# Associations of Alcohol Dehydrogenase and Aldehyde Dehydrogenase Polymorphism With Cognitive Impairment Among the Oldest-Old in China

**DOI:** 10.3389/fnagi.2021.710966

**Published:** 2022-02-25

**Authors:** Xurui Jin, Tingxi Long, Huashuai Chen, Yi Zeng, Xian Zhang, Lijing Yan, Chenkai Wu

**Affiliations:** ^1^Global Health Research Center, Duke Kunshan University, Kunshan, China; ^2^MindRank AI Ltd., Hangzhou, China; ^3^Center for the Study of Aging and Human Development and Geriatrics Division, Medical School of Duke University, Durham, NC, United States; ^4^Center for Healthy Aging and Development Studies, National School of Development, Peking University, Beijing, China; ^5^School of Health Sciences, Wuhan University, Wuhan, China; ^6^The George Institute for Global Health, Beijing, China

**Keywords:** ethanol intake, Asian flush, alcohol metabolizing genes, Alzheimer’s disease, cognitive impairment

## Abstract

Recent literature suggested that *ALDH2* mutation is associated with alcohol metabolism, and ethanol intake might jointly increase the risk of Alzheimer’s disease (AD) in mice. However, it is unclear whether this synergistic effect exists among humans. We examined the associations of four single nucleotide polymorphisms (SNPs) on aldehyde dehydrogenase (*ALDH*) and alcohol dehydrogenase (*ADH*) genes (i.e., *ALDH2* rs671, *ADH1B* rs1229984, *ADH1B* rs1042026, and *ADH1C* rs1693482) and cognitive impairment among the oldest-old. We also investigated whether this association was modified by ethanol intake from alcohol consumption. Data were from the Chinese Longitudinal Healthy Longevity Survey genetic sub-study, including 1,949 participants aged over 90 years. Participants with a Mini-Mental State Examination (MMSE) score of < 18 were considered cognitively impaired. Alcohol consumption was categorized as heavy, moderate, or never drinkers. With the dominant model, carrying A allele on rs671, C allele on rs1229984, and T allele on rs1042026 was associated with 33% (95% confidence interval [CI]: 5%, 69%), 33% (95% CI: 2%, 75%), and 29% (95% CI: 3%, 62%) higher odds of cognitive impairment in the multivariable-adjusted logistic model, respectively. We did not observe a significant interaction between those SNPs and alcohol consumption. Among the oldest-old, carrying *ALDH2* rs671 mutation was associated with higher odds of cognitive impairment independent of alcohol consumption.

## Introduction

Aging is a major contributor to cognitive decline and dementia across the world, and it is becoming a major public health concern. Previous studies have discussed demographic or socioeconomic risk factors for dementia among old adults, with limited attention on genetic risk factors using large samples. Most of the genetic research on cognitive function focused on candidate genes which have been demonstrated to be associated with Alzheimer’s disease (AD) or identified by genome-wide association studies (GWAS). Those candidate genes include apolipoprotein E (APOE), catechol-*O*-methyltransferase (COMT) (Komulainen et al., 2008), brain-derived neurotrophic factor (BDNF) (Bray et al., 2005), and dystrobrevin-binding protein 1 (DTNBP1) (Wray et al., 2008), with the *APOE* ε4 allele being by far the strongest genetic risk factor and accounts for about 5% of the variance in lifetime cognitive change and 4% of the variance in AD. In addition to those genetic factors, some recent works have also indicated the key role of some alcohol metabolism-related genes as enzymes involved in the detoxification of the ethanol metabolism in the pathology of AD.

Alcohol dehydrogenase (*ADH*) and aldehyde dehydrogenase (*ALDH*) are the two major alcohol-metabolizing enzymes (Chen et al., 2014). The mutation of several single nucleotide polymorphisms (SNPs) on those two genes results in the change of enzymatic activity, such as rs671 in *ALDH*, rs1693482 in *ADH1C*, and rs1229984 and rs1042026 in *ADH1B* (Hurley and Edenberg, 2012). Among the Asian population, the prevalence of *ADH1B*, *ADH1C*, and *ALDH2* mutation is performed. It is estimated that nearly 30% of people in Asia (∼8% of the world population) carry the genetic variants of the *ALDH2* A allele by rs671 (Eng et al., 2007). Previous literature has indicated that a reduced activity of *ADH* and *ALDH* would lead to an excess of acetaldehyde and result in oxidative stress and mitochondrial dysfunctions which have been identified in both familial and sporadic AD (Zhang and Ren, 2011; Chen et al., 2012; Swerdlow, 2018). At the population level, *ADH* and *ALDH* genes have been shown to be associated with alcohol dependence (Macgregor et al., 2009)—a leading cause of dementia (Koch et al., 2019). At present, the associations between *ALDH2* and *ADH* genetic polymorphisms with cognitive function or AD were inconclusive. Some studies suggested that *ALDH2* (rs671 polymorphism) is a risk factor for AD in Japanese (Kamino et al., 2000), whereas others reported no association. A recent study including 339 AD patients and 168 healthy controls investigated the association of several SNPs on *ADH* and *ALDH* with AD and found a suggestive association between *ADH1C* rs2241894 and AD among women (Wu et al., 2021). However, some other genetic studies have found that the negative impact of genetic factors may be cumulative by age, such as *APOE* gene on cognitive function, and there is a fewer study of *ADH* and *ALDH* genes among the oldest-old population.

A recent experimental study suggested that chronic excessive ethanol intake and the *ALDH2* gene mutation might jointly increase the risk of AD in mice (Joshi et al., 2019). Alcohol consumption, as one of the major risk factors of brain damage, is associated with the incidence of dementia. There is a J-shaped association of alcohol consumption with dementia, where excessive alcohol intake or abstinence increased dementia risk, compared with consuming 9–112 g/week (Topiwala and Ebmeier, 2018). In a large cross-sectional study from Southern China, occasional rather than moderate alcohol use was found to be associated with better cognitive function (Au Yeung et al., 2011), suggesting that the observed effects could be driven by a complex interaction between alcohol and other factors. In systematic reviews, drinking patterns are associated with AD and also cognitive function. It is unclear whether *ALDH2* mutation is associated with cognitive function and whether this mutation and ethanol intake synergistically contribute to the development of AD among humans.

The aim of the present study was twofold. First, we investigated the associations of four SNPs (i.e., *ALDH2* rs671, *ADH1B* rs1229984, *ADH1B* rs1042026, and *ADH1C* rs1693482) and cognitive function among nearly 2,000 Chinese oldest-old from a population-based cohort study. Considering the high prevalence of *ADH* and *ALDH* gene mutation in the Chinese population and their unique characterization, we then investigated whether this association would be modified by the level of alcohol consumption.

## Materials and Methods

### Participants

The present study used data from the CLHLS, which is an ongoing longitudinal study that began in 1998 with follow-up surveys for every 2–3 years. The CLHLS is a Chinese nationwide survey conducted in randomly selected counties and cities in 22 of 30 provinces covering 85% of the population of China. All centenarians from the selected areas who agreed to participate were included in the study. Based on sex and place of residence (i.e., living in the same street, village, city, or county) for a given centenarian, randomly selected octogenarians and non-agenarians were also sampled. More details about the sampling procedure and quality of data of this survey have been published elsewhere (Zeng et al., 2017). Ethical approval was obtained from the Research Ethics Committees of Peking University and Duke University (IRB00001052-13074). All participants or their legal representatives signed written consent forms in the baseline and follow-up surveys. In this cross-sectional study, we derived the data from the baseline survey of each participant. The analyses were based on the CLHLS genetic dataset, comprising 1,949 adults aged over 90 years.

### Cognitive Function

The cognitive function of CLHLS participants was assessed by the Chinese version of the Mini-Mental State Examination (MMSE) through a home-based interview, which includes 24 items, covering 7 subscales including orientation (4 points for time orientation and 1 point for place orientation); naming foods (naming as many kinds of food as possible in 1 min, 7 points); registration of 3 words (3 points); attention and calculation (mentally subtracting 3 iteratively from 20, 5 points); copy a figure (1 point); recall (delayed recall of the 3 words mentioned above, 3 points); and language (2 points for naming objectives, 1 point for repeating a sentence, and 3 points for listening and following directions). The MMSE score ranges from 0 to 30. Higher scores represent a better cognitive function. The validity and reliability of this Chinese MMSE have been verified in several previous studies (Zhang, 2006; An and Liu, 2016). Consistent with previous studies (Zhang et al., 2019), because a high proportion of our participants did not have formal education (∼70%), cognitive impairment was defined as an MMSE score of < 18.

### Alcohol Consumption and Other Covariates

During each interview, the interviewers measured a range of demographic, behavioral, and socioeconomic covariates. Alcohol consumption was self-reported and categorized as heavy, moderate, or never drinkers. Current alcohol users who consumed >25 g of alcohol per day (for men) and > 15 g (for women) were considered heavy drinkers. Current alcohol users who consumed ≤25 g (for men) and ≤15 g (for women) were considered moderate drinkers. Participants who reported they never drank were considered never drinkers. All variables were measured at baseline (indicate year). All self-reported information was collected through face-to-face home interview by trained research staff members with more than 12 years of education.

Following the previous studies, we included the following variables as confounders: age (years), sex, residence (rural vs. urban), education years, smoking (never, former, and current), regular physical activity (yes vs. no), dietary pattern, leisure activity score, and self-reported chronic diseases [e.g., hypertension, diabetes, heart disease, stroke, and chronic obstructive pulmonary disease (COPD)]. Dietary pattern was categorized as unfavorable, intermediate, or favorable by a simplified healthy eating index based on the intake frequency of nine food categories, including fruits, vegetables, fish, bean products, tea, garlic, eggs, sugar, and salt-preserved vegetables. A 3-point scale question was used to measure the current intake frequency of each food group: “always or almost every day,” “sometimes or occasionally,” or “rarely or never.” Those 3 terms received the scores of 2, 1, or 0, respectively, with higher scores indicating a higher level of consumption. Two of those food groups, i.e., sugar and salt-preserved vegetables, were received the scores of 0, 1, and 2, respectively, for some evidence of the negative impact of the high consumption of those two food groups. The leisure activity score was summarized from the following eight activities: visiting neighbors, shopping, cooking, washing clothes, walking 1 km, lifting 5 kg, crouching and standing up three times, and taking public transportation. We scored each activity as 1 for “never,” 2 for “sometimes,” and 3 for “almost every day.” The score ranged from 5 to 21 with a higher score, indicating more leisure activities. All self-reported information was collected through face-to-face home interview by trained research staff members at the baseline survey. Interviewees were encouraged to answer as many questions as possible. If they were unable to answer questions, a close family member or another proxy, such as a primary caregiver, provided answers (Zeng, 2012).

### Genotyping

The CLHLS collected DNA samples from parts of participants in 1998, 2000, 2002, 2005, 2008–2009, and 2011–2012 waves of the survey. Genotyping of DNA samples was produced by the Beijing Genomics Institute (BGI), and the BGI genotyping quality control procedures of the CLHLS genetic study have been published elsewhere (Zeng et al., 2016). We extracted four SNPs associated with alcohol metabolism from the GWAS data (i.e., *ALDH2* rs671, *ADH1B* rs1229984, *ADH1B* rs1042026, and *ADH1C* rs1693482).

The genotypes were defined by following the additive and dominant models. In a dominant model, any genotype that contains one or two copies of the minor allele is coded as one; otherwise, the genotype that does not contain any copy of the minor allele is coded as zero. In an additive model, carrying two copies of the minor allele was coded as three. One copy of the minor allele with one copy of the major allele and two major alleles were coded as two and one, respectively. We mainly used the dominant models in the GxE analysis to define the genotype because it can distinguish the genotype carriers and non-carriers, but the additive model cannot. In addition, further grouping the samples in the additive model would result in many more GxE interaction terms in the regressions and would, in turn, negatively affect the estimates and complicate the discussions.

### Statistical Analysis

The characteristics of participants were compared according to cognitive function (with vs. without impairment). Means and standard deviations (SDs) were calculated for continuous variables; counts and percentages were calculated for categorical variables. We used both logistic regression (binary outcome: cognitive impairment) and linear regression (continuous outcome: MMSE score) models to examine the unadjusted and adjusted associations between the four polymorphisms and cognitive impairment, respectively. We build two regression models, namely, partly adjusted: adjusted for age at baseline in years, sex, residency, and education years; and fully adjusted: additionally adjusted for smoking status, alcohol consumption, current physical activity, dietary pattern and leisure activity score, and five kinds of self-reported diseases on the basement of the partly adjusted model.

To examine whether the association between the four polymorphisms and cognitive impairment was modified by alcohol consumption, we first ran a logistic regression model with an interaction term between those four polymorphisms and alcohol consumption, respectively, and then conducted the regression analysis among persons with different levels of alcohol consumption (e.g., heavy, moderate, and never drinkers), separately.

All statistical analyses were conducted using STATA version 14.0 (Stata Corp., College Station, TX, United States).

## Results

### Study Sample Characteristics

Demographic and the four polymorphisms information are detailed by cognitive function in Table 1. A total of 1,949 participants were included after excluding 101 participants who lacked cognitive assessment (*n* = 64) and aged below 90 years (*n* = 37). Of 1,949 participants aged over 90 years, the average age was 101.3 ± 3.3 years; 76.3% were women. Participants with cognitive impairment are more likely to be older, women, with lower education level, not smoking, with unfavorable dietary pattern, and with lower leisure activities (Table 1, *p*-values < 0.05).

**TABLE 1:** Selected characteristics of the participants by cognitive function.

Characteristics^a^		Cognitive impairment^a^		*P*-value
	Total *N* = 1,949	Without *N* = 805	With *N* = 1,144	

**Count (%) unless otherwise indicated**				
Age, mean ± SD	101.3 (3.3)	100.9 (3.3)	101.5 (3.2)	< 0.001
**Sex**				< 0.001
Women	1487 (76.3)	540 (67.1)	947 (82.8)	
Men	462 (23.7)	265 (32.9)	197 (17.2)	
MMSE score, mean ± SD	14.2 (10.4)	24.9 (3.4)	6.76 (6.4)	< 0.001
**Residence**				0.081
Urban	719 (36.9)	323 (40.1)	396 (34.6)	
Rural	1230 (63.1)	482 (59.9)	748 (65.4)	
Education years, mean ± SD	0.93 (2.5)	1.5 (3.2)	0.57 (1.8)	< 0.001
**Alcohol consumption**				0.053
Never drink	1513 (77.6)	604 (75.0)	909 (79.5)	
Moderate drink	199 (10.2)	88 (10.9)	111 (9.7)	
Heavy drink	237 (12.2)	113 (14.0)	124 (10.8)	
**Smoking**				0.026
Never	1712 (90.6)	828 (89.0)	884 (92.2)	
Former	148 (7.8)	82 (8.8)	66 (6.9)	
Current	29 (1.5)	20 (2.2)	9 (0.9)	
**Dietary pattern^b^**				< 0.001
Unfavorable	804 (41.3)	261 (32.4)	543 (47.5)	
Intermediate	634 (32.5)	273 (33.9)	361 (31.6)	
Favorable	511 (26.2)	271 (33.7)	240 (21.0)	
**Regular physical activity**				0.05
Yes	522 (27.8)	310 (33.4)	212 (22.2)	
No	1359 (72.2)	617 (66.6)	742 (77.8)	
Leisure activity score^b^	9.6 (2.4)	10.6 (2.5)	8.7 (1.9)	< 0.001
***ALDH2*, rs671^c^**				0.083
GG	527 (65.5)	733 (64.1)	1260 (64.6)	
AG	241 (29.9)	359 (31.4)	600 (30.8)	
AA	37 (4.6)	52 (4.5)	89 (4.6)	
***ADH1B*, rs1229984**				0.315
TT	495 (61.5)	639 (55.9)	1134 (58.2)	
CT	254 (31.6)	431 (37.7)	685 (35.1)	
CC	56 (7.0)	74 (6.5)	130 (6.7)	
***ADH1B*, rs1042026**				0.328
CC	516 (64.1)	683 (59.7)	1199 (61.5)	
CT	244 (30.3)	398 (34.8)	642 (32.9)	
TT	45 (5.6)	63 (5.5)	108 (5.5)	
***ADH1C*, rs1693482**				0.323
CC	694 (86.2)	958 (83.7)	1652 (84.8)	
CT	103 (12.8)	177 (15.5)	280 (14.4)	
TT	8 (1.0)	9 (0.8)	17 (0.9)	

*MMSE, Mini-Mental State Examination.*

*^a^Cognitive impairment: cognitive impairment was defined by an MMSE score of < 18.*

*^b^Dietary score: the score was calculated from the frequency for intake of nine foods: fruits, vegetables, fish, bean products, tea, garlic, eggs, sugar, and salt-preserved vegetables. For two of 9 variables, i.e., sugar and salt-preserved vegetables, the answer of “always or almost every day,” “sometimes or occasionally,” or “rarely or never” received the scores of 0, 1, or 2, respectively; for the other 7 variables, the same three responses received the scores of 2, 1, or 0, respectively. Scores for the 9 variables were then summed to obtain a scale ranging from 0 to 18 with higher scores indicating higher frequency for fruits, vegetables, fish, bean products, tea, garlic, and eggs, while higher scores meant lower frequencies for sugar and salt-preserved vegetables. The dietary pattern was defined by the trisection of the dietary pattern score (lowest trisection: unfavorable, intermediate: intermediate, highest: favorable).*

*^c^Leisure activity score: eight activities were assessed: visiting neighbors, shopping, cooking, washing clothes, walking 1 km, lifting 5 kg, crouching and standing up three times, and taking public transportation. We scored each activity as 1 for “never,” 2 for “sometimes,” and 3 for “almost every day.” The score ranged from 5 to 21 with higher score indicating more leisure activities.*

### Association of Cognitive Function With *ADH* and *ALDH2* Genes

In the partially adjusted logistic regression model, carrying A allele on rs671, C allele on rs1229984, T allele on rs1042026, and T allele on rs1693482 was associated with higher odds of cognitive impairment [rs671: odds ratio (OR): 1.19, 95% CI: 0.97, 1.47; rs1229984: OR: 1.27, 95% CI: 1.00, 1.61; rs1042026: OR: 1.26, 95% CI: 1.04, 1.55; rs1693482: OR: 1.19, 95% CI: 0.91, 1.57; Table 2]. In the fully adjusted logistic regression model, the associations of rs671, rs1229984, and rs1042026 with cognitive impairment persisted to be significant. In the partially adjusted linear regression model, carrying T allele on rs1042026 and T allele on rs1693482 was significantly associated with a lower MMSE score of 0.90 (95% CI: 0.01, 1.80). In the fully adjusted linear regression model, carrying A on rs671 was associated with a lower MMSE score of 1.05 (95% CI: −2.03, −0.09).

**TABLE 2:** Association of *ALDH2* rs671, *ADH1B* rs1229984, *ADH1B* rs1042026, and *ADH1C* rs1693482 polymorphisms with cognitive impairment.^a^

Independent variable	Logistic regression OR of cognitive impairment^a^, (95% CI)		Linear regression Coefficient for MMSE score (95% CI)	
	Partially adjusted^b^	Fully adjusted^c^	Partially adjusted	Fully adjusted **
**Additive model**				
rs671 GG vs. AG	1.19 (1.00, 1.47) **	1.36 (1.07, 1.74) **	−0.61 (−1.62, 0.40)	−1.23 (−2.22, −0.26) **
rs671 GG vs. AA	1.02 (0.64, 1.64)	1.12 (0.66, 1.90)	1.25 (−0.99, 3.51)	0.84 (−1.32, 3.00)
rs1229984 TT vs. CT	1.30 (1.06, 1.60) **	1.32 (1.05, 1.67) **	−1.11 (−2.09, −0.13) **	−1.02 (−1.97, −0.08) **
rs1229984 TT vs. CC	1.25 (0.83, 1.88)	1.09 (0.70, 1.72)	−0.17 (−2.13, 1.78)	0.35 (−1.49, 2.21)
rs1042026 CC vs. CT	1.22 (1.00, 1.51) **	1.29 (1.03, 1.64) **	−0.82 (−1.82, 0.17) *	−0.92 (−1.87, −0.01) **
rs1042026 CC vs. TT	1.29 (0.83, 2.03)	1.05 (0.65, 1.72)	0.02 (−2.11, 2.16)	0.99 (−1.04, 3.04)
rs1693482 CC vs. CT	1.22 (0.93, 1.62) *	1.16 (0.84, 1.59)	−1.39 (−2.71, −0.08) **	−1.27 (−2.55, −0.004) **
rs1693482 CC vs. TT	0.88 (0.31, 2.56)	1.07 (0.34, 3.28)	2.54 (−2.58, 7.68)	1.39 (−3.50, 6.28)
**Dominant model**				
rs671 GG vs. AG/AA	1.19 (0.97, 1.47) *	1.33 (1.05, 1.69) **	−0.53 (−1.49, 0.42) *	−1.05 (−2.03, −0.09) **
rs1229984 TT vs. CT/CC	1.27 (1.00, 1.61) **	1.33 (1.02, 1.75) **	−0.90 (−1.80, −0.01) **	−0.93 (−2.00, 0.12) *
rs1042026 CC vs. CT/TT	1.26 (1.04, 1.55) **	1.29 (1.03, 1.62) **	−0.76 (−1.69, 0.17)	−0.70 (−1.63, 0.23)
rs1693482 CC vs. CT/TT	1.19 (0.91, 1.57) *	1.15 (0.84, 1.57)	−1.19 (−2.42, 0.05) *	−1.09 (−2.35, 0.15) *

Significance: *p < 0.1, **p < 0.05.

*^a^Defined as a Mini-Mental State Examination score of < 18.*

*^b^Model was adjusted for age at baseline in years, sex, residency, and education years.*

*^c^Model was additionally adjusted for smoking status, alcohol consumption, current physical activity, dietary index, and leisure activity score (continuous) and five kinds of self-reported diseases on the basement of partly adjusted model.*

In the additive model, the one major allele with one minor allele genotypes of rs671, rs1229984, and rs1042026 was significantly associated with lower MMSE scores and higher odds of cognitive impairment compared with the two major allele genotypes (Table 2). However, the two minor allele genotypes of those four SNPs were not significantly associated with MMSE score or cognitive impairment in the fully or partially adjusted model.

### Association Between Alcohol Consumption and Cognitive Function

The association between alcohol consumption and cognitive impairment was analyzed in the partially adjusted model and fully adjusted model (Supplementary Table 1). In the fully adjusted linear regression model, moderate alcohol use and heavy alcohol use were significantly associated with a lower MMSE score (moderate: −2.52, 95% CI: −2.49, −2.55; heavy: −2.83, 95% CI: −2.36, −3.30) compared with participants who never consumed alcohol.

### Subgroup Analyses by Alcohol Consumption

Table 3 presents the associations between four SNPs (those specified here) with the dominant model and cognitive impairment stratified by alcohol consumption. In the subgroup analyses, there is no evidence that the association of each of four SNPs with cognitive impairment differed by alcohol consumption (*p-*values for interactions > 0.05).

**TABLE 3:** The association of *ALDH2* rs671 polymorphism with cognitive impairment stratified by alcohol consumption and fresh fruit consumption.^a^

	Participants (N)	Odds ratio (95% Confidence Interval)^b^	*P* for interaction
**Alcohol consumption**			
**Never**			
rs671 GG vs. AG/AA	912/601	1.25 (0.97, 1.62)	
rs1229984 TT vs. CT/CC	889/624	1.36 (1.05, 1.75)	
rs1042026 CC vs. CT/TT	937/576	1.38 (1.07, 1.79)	
rs1693482 CC vs. CT/TT	1295/218	1.39 (0.95, 2.03)	
**Moderate drink**			
rs671 GG vs. AG/AA	146/53	1.45 (0.63, 3.34)	0.58
rs1229984 TT vs. CT/CC	121/78	0.97 (0.47, 2.02)	0.44
rs1042026 CC vs. CT/TT	131/68	0.77 (0.36, 1.63)	0.15
rs1693482 CC vs. CT/TT	167/32	0.47 (0.19, 1.23)	0.079
**Heavy drink**			
rs671 GG vs. AG/AA	203/34	2.14 (0.84, 5.50)	0.26
rs1229984 TT vs. CT/CC	124/113	1.13 (0.63, 2.13)	0.63
rs1042026 CC vs. CT/TT	131/106	1.01 (0.54, 1.90)	0.51
rs1693482 CC vs. CT/TT	190/47	0.85 (0.39, 1.87)	0.54

*^a^Defined as a Mini-Mental State Examination score of < 18.*

*^b^From logistic regression with adjustments for age at baseline in years, sex, residency, education years, smoking status, alcohol consumption, current physical activity, dietary index, and leisure activity score (continuous) and five kinds of self-reported diseases on the basement of partly adjusted model.*

## Discussion

Using data from nearly 2,000 Chinese adults aged over 90 years, we found that the SNPs on *ADH* and *ALDH2* genes were associated with higher odds of cognitive impairment. In addition, the results showed that the associations between those polymorphisms and cognitive impairments were not modified by alcohol consumption, which is different from the findings of a recent experimental study (Joshi et al., 2019).

The results indicated that carrying the *ALDH2* and *ADH* mutation was associated with higher odds of cognitive impairment among the oldest-old population. Its mutation results in a reduction of the *ALDH2* enzymatic activity, which is widely mutated in the Asian population (∼30%). One possible explanation was that those two mutations have a negative impact on mitochondrial functions and the metabolism of 4-hydroxy-2-nonenal (4-NHE). *ALDH2* and *ADH* are involved in the detoxification of the ethanol metabolism and other aldehydes, including 4-HNE. It has been shown that 4HNE concentration increased in the brain tissue of the ALDH2*2 transgenic mice in an age-dependent manner. Such increases correlated with neurodegeneration memory loss and AD-like pathological changes in these ALDH2*2 transgenic mice (Ohsawa et al., 2008). In addition, 4HNE levels are higher in the hippocampus of postmortem samples from patients with AD (Williams et al., 2006). Among the population level, previous studies reported the inconsistent results on this association. A cross-sectional case-control study has shown that *ALDH2* genotype is associated with cognitive function among 139 Chinese patients with Parkinson’s disease (mean age: 63.0 years) (Yu et al., 2016), and another study has demonstrated its association among 411 Chinese with an average age of 77.4 years (Wang et al., 2008). However, in another study including 690 Korean community residents (mean age: 72.8 years) (Kim et al., 2004), the association between *ALDH2* genotype and cognitive functions was not significant. This paradox finding may be due to the mean age difference of study samples. It is plausible that the genetic risk of carrying the *ALDH2* mutation is cumulative (Licher et al., 2019). Researchers found that *APOE* as well as other common genetic variants could have a cumulative risk on the progression of dementia and AD as age advances (van der Lee et al., 2018). To be more specific, with the negative influence of those mutations on neuronal functioning, the carriers may have a higher speed of neuronal cell loss which is irreversible. Accordingly, during the early life stage, the difference in cognitive function between the carriers and non-carriers may not be significant, while as people age, the harmful effects of genetic variants may build up, and the carriers may have a lower average cognitive function compared with non-carriers of the same age due to the cumulative damage on neurons (Bai and Mei, 2011). In addition to rs671 on *ALDH2*, we also found that rs1229984 and rs1042026 on *ADH1B* gene were associated with the cognitive function among the oldest-old population. The *ADH1B* rs1229984 T allele encodes a super active enzyme subunit that could accelerate the conversion from ethanol into acetaldehyde (Lin et al., 2021). *ADH1C* rs1693482 is found to be associated with a twofold difference in *ADH* Vmax *in vitro* (Birley et al., 2009). One experimental study has found that in the animal model of AD, the overexpressing of *ADH1B* suppresses the β-amyloid-induced neuron apoptosis (Wang et al., 2019). Corresponding the results, it may indicate that the genes associated with alcohol metabolism might be important in the progress of cognitive impairment or AD. To note, these results also determined the potential role of mitochondrial dysfunctions in the pathology of AD.

We found that those associations were not modified by alcohol consumption. One experimental study found that the ALDH2*2 mutation increases the damage by sustained ethanol exposure in mouse brain, and they found that ALDH2*2 deficiency increases ethanol-induced neuroinflammation *in vivo* (Joshi et al., 2019). However, we found that on the population level, such interactive effect was not significant. It may indicate that the damage of alcohol to the brain is stable and consistent which may not be varied by genotype. In addition, another possible explanation was that the consumption of stored fruits and vegetables, as well as fruit juice, would bring ethanol into the human body and the total ethanol intake is more difficult to measure.

Methodological strengths of this study include a large sample size of the oldest old, and we have included multiple genes related to alcohol metabolism. This study also has several limitations: (1) it has a cross-sectional design and cannot evaluate changes in alcohol consumption or establish causality. However, the results were robust to adjustment of a number of indicators such as diseases. Nevertheless, prospective studies on the incidence of cognitive impairment are warranted; (2) the alcohol consumption was self-reported, thus non-specific (Muntner et al., 2014). This possibility of recall bias is a common concern in the longitudinal cohort studies, although CLHLS data have been validated as being reliable in previous studies (Zeng et al., 2008). In the CLHLS study, all self-reported information was collected through face-to-face home interview by trained research staff members. Interviewees were encouraged to answer as many questions as possible. If they were unable to answer questions, a close family member or another proxy, such as a primary caregiver, provided answers (Zeng et al., 2008). (3) We used the Chinese version of MMSE to measure cognition, which is not a clinical diagnosis for cognitive impairment (Zhang, 1993; Chou, 2003). However, it is a validated instrument in population-based studies. (4) As the study included only participants who aged over 90 years and the average lifespan of women is longer than men, a higher proportion of women were taken into the analysis sample which may lead to bias. (5) The percentage of drinker is 20% and the results need to be validated within a larger cohort.

## Conclusion

We found that some SNPs associated with alcohol metabolism were associated with higher odds of cognitive impairment among the Chinese oldest-old and those associations were independent of alcohol consumption. Due to its mutation can be easily observed even without genotyping, it may have more public health implications especially for the Asian population on the AD risk stratification and alcohol control. Further studies on how to modify the genetic risk for cognitive impairment associated with gene mutation associated with alcohol metabolism are warranted.

## Data Availability Statement

Publicly available datasets were analyzed in this study. This data can be found here: https://sites.duke.edu/centerforaging/programs/chinese-longitudinal-healthy-longevity-survey-clhls.

## Ethics Statement

The studies involving human participants were reviewed and approved by the Biomedical Ethics Review Committee of Peking University (IRB00001052-11015). The patients/participants provided their written informed consent to participate in this study.

## Author Contributions

All authors listed have made a substantial, direct, and intellectual contribution to the work, and approved it for publication.

## Conflict of Interest

XJ and XZ were employed by MindRank AI Ltd. The remaining authors declare that the research was conducted in the absence of any commercial or financial relationships that could be construed as a potential conflict of interest.

## Publisher’s Note

All claims expressed in this article are solely those of the authors and do not necessarily represent those of their affiliated organizations, or those of the publisher, the editors and the reviewers. Any product that may be evaluated in this article, or claim that may be made by its manufacturer, is not guaranteed or endorsed by the publisher.
