# Supplementary material for: Associations of Alcohol Dehydrogenase and Aldehyde Dehydrogenase Polymorphism With Cognitive Impairment Among the Oldest-Old in China
Source: Front Aging Neurosci. 2022 Feb 25;13:710966. doi: 10.3389/fnagi.2021.710966 (PMC8965653; doi:10.3389/fnagi.2021.710966)
Supplement: Supplementary file 1 [file Table_1.docx]

**Appendix**

**Table S1**

| **Independent variable** | **Logistic regression**  **OR of cognitive impairment ^a^, (95% CI)** | | **Linear regression**  **Coefficient for MMSE score , (95% CI)** | |
| --- | --- | --- | --- | --- |
|  | **Partially adjusted ^b^** | **Fully adjusted ^c^** | **Partially adjusted** | **Fully adjusted ^**^** |
| **One-year increase in Age** | 1.07 (1.06, 1.10) | 1.05 (1.02, 1.08) | -0.30 (-0.36, -0.25) | -0.11 (-0.17, -0.05) |
| **Gender** |  |  |  |  |
| Women | Ref. | Ref. | Ref. | Ref. |
| Man | 0.57 (0.45, 0.73) | 0.58 (0.44, 0.79) | 2.28 (1.11, 3.46) | 1.95 (0.78, 3.11) |
